# Presence of anti-rituximab antibodies predicts infusion-related reactions in patients with systemic lupus erythematosus

**DOI:** 10.1136/annrheumdis-2019-215200

**Published:** 2019-03-28

**Authors:** Chris Wincup, Madhvi Menon, Edward Smith, Ann Schwartz, David Isenberg, Elizabeth C Jury, Claudia Mauri

**Affiliations:** 1 Centre for Rheumatology, Division of Medicine, UCL, London, UK; 2 Bioanalysis, Immunogenicity & Biomarkers, IVIVT RD Platform Technology & Science, GlaxoSmithKline Plc, Philadelphia, Pennsylvania, USA

**Keywords:** systemic lupus erythematosus, treatment, dmards (biologic), b cells, autoantibodies

Rituximab (RTX) is a chimeric monoclonal anti-CD20 antibody used in the treatment of various rheumatic diseases.^1^ Although generally well tolerated, infusion-related reactions (IRR) represent the most common adverse event associated with treatment and are difficult to predict. In patients undergoing treatment for rheumatoid arthritis, the incidence of IRR is quoted as 3%–4%^2 3^; however, in systemic lupus erythematosus (SLE) this is significantly higher at 19%.^4^ To date, few studies have assessed the role antidrug antibodies (ADA) play in the lack of response or development of IRR to RTX in SLE. Here, we investigate how the presence of ADA relates to IRR and effectiveness of RTX therapy in SLE.

Fifty-seven patients fulfilling American College of Rheumatology criteria were recruited from the lupus clinic at University College London Hospital, UK. All patients were receiving RTX for active SLE (British Isles Lupus Assessment Group [BILAG] A or 2B scores) for the first time. Confirmed IRR were recorded in electronic health records. Baseline characteristics including complement C3 (C3), double-stranded DNA antibody titres (dsDNA) and BILAG score were recorded at the time of treatment and at each subsequent clinic visit. CD19 positive lymphocyte (CD19) levels were measured at 1 and 6 months following treatment. IRR were classified in accordance with Common Terminology Criteria for Adverse Events v4 criteria (online supplementary table 1).^5^ Presence of ADA was assessed via a bridging electrochemiluminescence assay using biotinylated and ruthenylated RTX as capture and detection. Χ^2^ with Bonferroni correction was used to compare categorical differences between ADA^+^ and ADA^-^ groups. Paired t-test was used to assess for differences immediately prior to and at 6 months following treatment.

10.1136/annrheumdis-2019-215200.supp1 Supplementary data

As shown in table 1, ADA were identified in 37% of patients following treatment. ADA^+^ patients were younger both at diagnosis (p=0.03) and at the time of first treatment with RTX (p<0.001). In spite of low overall numbers, ADA were more commonly seen in males (p=0.04). There was no significant difference in concomitant treatment, disease manifestation and ethnicity. At the time of treatment, there was no difference in C3, dsDNA titres or BILAG. Figure 1 demonstrates that at 6 months post-treatment, ADA^-^ patients show a significant increase in C3 levels (p=0.003) and reduction in dsDNA antibody binding (p=0.008) in keeping with effective response to treatment. In ADA^+^ patients, although normalisation of C3 was seen at 6 months (p=0.007), there was no observed improvement in dsDNA titres (p=0.96). Both ADA^+^ and ADA^-^ patients displayed a significant improvement in global BILAG score 6 months after treatment (p<0.0001). There was no difference in CD19 between ADA^+^ and ADA^-^ patients at either 1 or 6 months post-treatment. Of the 57 patients recruited, 25 patients underwent retreatment with RTX (18 ADA^+^ and 7 ADA^-^ patients). All ADA^+^ patients developed IRR, whereas no IRR was reported in those who were ADA^-^ (p<0.001). Severe reactions resulting in hospitalisation were seen in three cases in which ADA titres were >1500 IU. In one such case, subsequent treatment with ofatumumab (a fully humanised anti-CD20 monoclonal antibody) was well tolerated without the occurrence of further IRR.

**Figure 1:**
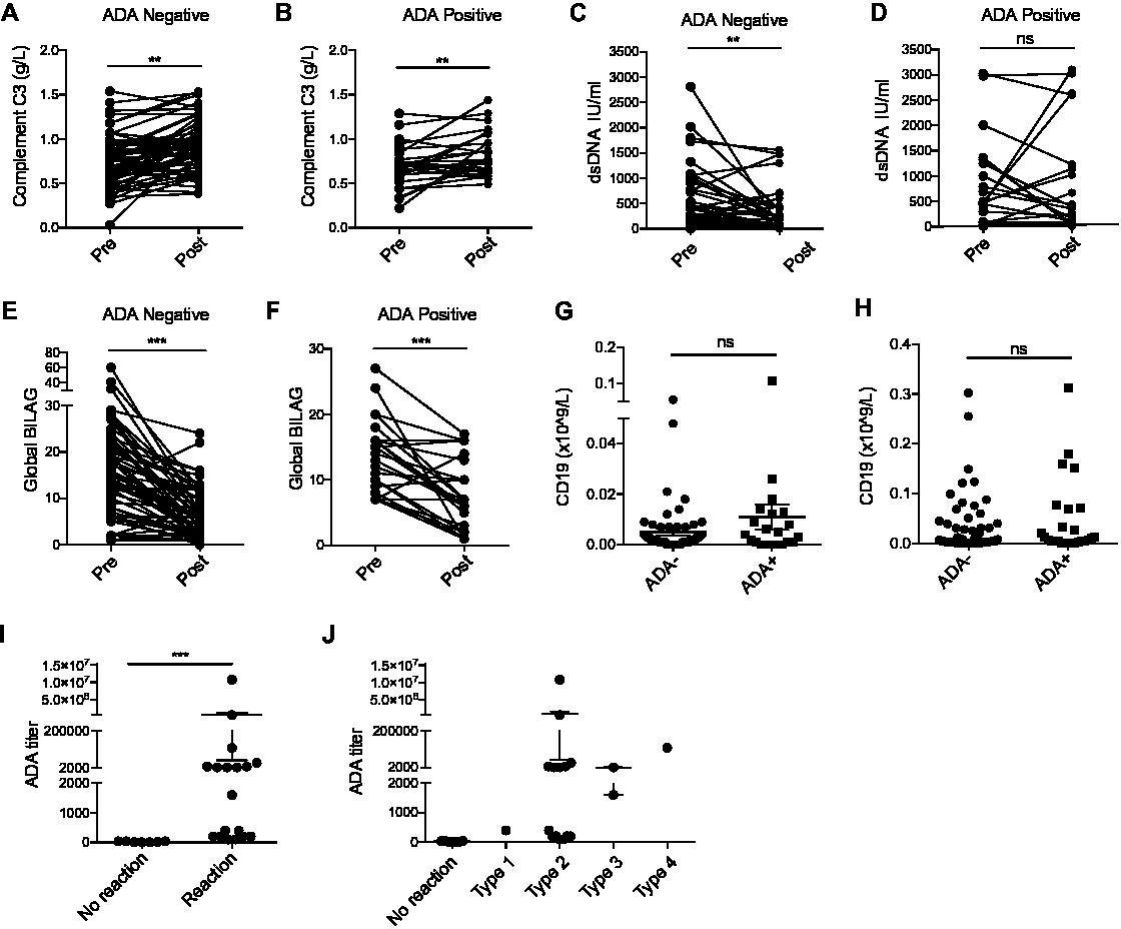
Complement C3 levels significantly improve at 6 months post-treatment with RTX in ADA^-^ (A) and ADA^+^ patients (B). Although a significant improvement in dsDNA binding was seen in in those who are ADA^-^ at 6 months post-treatment (p=0.008) (C), this was not seen in those who were ADA^+^ (p=0.96) (D). A significant improvement in global BILAG score was seen in both (E) ADA^-^ (p<0.0001) and (F) ADA^+^ patients (p<0.0001). No significant difference in CD19 positive lymphocyte count was seen at either 1 (G) and 6 (H) months post-treatment regardless of ADA status. ADA^+^ patients undergoing retreatment with RTX all developed IRR (I). The most common type of reaction observed was CTCAE type 2 with more severe reactions seen in those with higher ADA titres (J). ADA, antidrug antibody; CTCAE, Common Terminology Criteria for Adverse Events; IRR, infusion-related reactions; RTX, rituximab.

**Table 1:** Patient characteristics

	ADA positive N=21	ADA negative N=36	P value
Age at diagnosis, years (SD)	22.0 (7.9)	30.0 (11.8)	<0.001***
Age at time of treatment, years (SD)	31.9 (9.1)	42.9 (11.1)	0.03*
Disease duration, months (SD)	119 (76)	151 (108)	NS
Gender (Female:Male) (%)	16:5 (76:24)	34:2 (94:6)	0.04*
Ethnicity, n (%)			
** **Afro-Caribbean	5 (24)	6 (17)	NS
** **Caucasian	9 (43)	19 (53)	NS
** **South Asian	3 (14)	6 (17)	NS
** **East Asian	3 (14)	2 (5)	NS
** **Other	1 (5)	3 (8)	NS
Indication for treatment, n (%)			
** **Nephritis	10 (48)	7 (19)	NS
** **Arthritis	5 (24)	11 (31)	NS
** **Haematological	3 (14)	2 (6)	NS
** **Neuro-psychiatric	0 (0)	1 (3)	NS
** **Serositis	1 (5)	3 (8)	NS
** **Cutaneous	6 (29)	9 (25)	NS
** **Other	3 (14)	3 (8)	NS
Additional treatment, n (%)			
** **Hydroxychloroquine	10 (48)	20 (56)	NS
** **Azathioprine	5 (24)	5 (14)	NS
** **Mycophenolate mofetil	4 (19)	4 (11)	NS
** **Methotrexate	0 (0)	4 (11)	NS
** **Prednisolone	17 (81)	27 (75)	NS
** **Cyclophosphamide†	15 (71)	22 (61)	NS
Disease activity at the time of treatment			
** **Complement C3 (g/L) (SD)	0.76 (0.29)	0.69 (0.24)	NS
** **Anti-dsDNA titres (IU) (SD)	411.7 (651.6)	699.8 (874.2)	NS
** **Global BILAG score (SD)	15.8 (10.2)	13.5 (5.6)	NS
Disease activity 6 months post-treatment			
** **Complement C3 (g/L) (SD)	0.91 (0.27)	0.83 (0.24)	NS
** **Anti-dsDNA titres (IU) (SD)	201.4 (369.6)	682.0 (1009.0)	0.04*
** **Global BILAG score (SD)	6.4 (5.4)	7.4 (5.4)	NS

*p<0.05; ***p<0.001.

†Given in combination with rituximab therapy.

ADA, antidrug antibody;BILAG, British Isles Lupus Assessment Group; dsDNA, double-stranded DNA; NS, not significant.

We demonstrate that ADA to RTX are common in those undergoing treatment for SLE and have a clear association with subsequent IRR. Contrary to previous studies,^6^ our findings suggest that CD19 count is not affected by ADA, however the presence of ADA appeared to impair normalisation of dsDNA titres following treatment. If validated, these findings may support routine screening for ADA prior to treatment with RTX, thus potentially identifying patients at risk of developing IRR and prompting greater caution and enhanced surveillance. In the context of high ADA titres, this may necessitate the use of an alternate B-cell depleting agent (such as ofatumumab).

## References

[1] Turner-Stokes T, Lu TY, Ehrenstein MR, et al The efficacy of repeated treatment with B-cell depletion therapy in systemic lupus erythematosus: an evaluation. Rheumatology 2011;50:1401–8. 10.1093/rheumatology/ker018 21398661

[2] Salmon JH, Perotin JM, Morel J, et al Serious infusion-related reaction after rituximab, abatacept and tocilizumab in rheumatoid arthritis: prospective registry data. Rheumatology 2018;57:134–9. 10.1093/rheumatology/kex403 29069471

[3] Arredondo-Garza T, Majluf-Cruz A, Vela-Ojeda J, et al Peri-infusional adverse reactions to rituximab in patients with non-Hodgkin's lymphoma. Arch Med Res 2013;44:549–54. 10.1016/j.arcmed.2013.09.011 24120421

[4] Lan L, Han F, Chen JH Efficacy and safety of rituximab therapy for systemic lupus erythematosus: a systematic review and meta-analysis. J Zhejiang Univ Sci B 2012;13:731–44. 10.1631/jzus.B1200057 22949364 PMC3437371

[5] Can M, Alibaz-Öner F, Yılmaz-Öner S, et al Accelerated infusion rates of rituximab are well tolerated and safe in rheumatology practice: a single-centre experience. Clin Rheumatol 2013;32:87–90. 10.1007/s10067-012-2094-1 23053686

[6] Albert D, Dunham J, Khan S, et al Variability in the biological response to anti-CD20 B cell depletion in systemic lupus erythaematosus. Ann Rheum Dis 2008;67:1724–31. 10.1136/ard.2007.083162 18250115

