# Supplementary material for: Presence of anti-rituximab antibodies predicts infusion-related reactions in patients with systemic lupus erythematosus
Source: Ann Rheum Dis. 2019 Mar 28;78(8):1140–2. doi: 10.1136/annrheumdis-2019-215200 (PMC6691858; doi:10.1136/annrheumdis-2019-215200)
Supplement: Supplementary data [file annrheumdis-2019-215200supp001.docx]

**SUPPLEMENTARY TABLE S1:**

| Infusion reaction category | Description |
| --- | --- |
| Type 1 IRR | Mild transient reaction; infusion interruption not indicated; intervention not indicated |
| Type 2 IRR | Infusion interruption indicated but responds promptly to symptomatic treatment (e.g. anti-histamines, intravenous fluids, steroids, etc) |
| Type 3 IRR | Prolonged (e.g. not rapidly responsive to symptomatic medication and/or brief interruption of infusion); recurrence of symptoms following initial improvement, hospitalisation indicated for clinical sequelae |
| Type 4 IRR | Life-threatening consequences; urgent intervention indicated |
| Type 5 IRR | Death |

**SUPPLEMENTARY TABLE 1:** Common Terminology Criteria for Adverse Events (CTCAE v4) criteria for classifying adverse events to medication.
